# Bilateral herpetic keratitis presenting with unilateral neurotrophic keratitis in pemphigus foliaceus: a case report

**DOI:** 10.1186/1752-1947-5-328

**Published:** 2011-07-27

**Authors:** Hee K Yang, Young K Han, Won R Wee, Jin H Lee, Ji W Kwon

**Affiliations:** 1 Department of Ophthalmology, Seoul National University College of Medicine, Seoul Artificial Eye Center, Seoul National University Hospital Clinical Research Institute, 28 Yongon-dong, Chongno-gu, Seoul, 110-744, Korea; 2 Department of Ophthalmology, Seoul National University Boramae Hospital, Shindaebang-dong, Dongjak-gu, Seoul, 156-707, Korea; 3 Department of Ophthalmology, Myongji Hospital, Kwandong University College of Medicine, 697-24, Hwajung-Dong, Deokyang-Gu, Goyang-Si, Gyeonggi-Do, 112-270, Korea

## Abstract

**Introduction:**

We report a case of bilateral herpetic keratitis developing after rapid oral corticosteroid tapering in a patient with pemphigus foliaceus, which was followed by unilateral neurotrophic keratitis that was treated with amniotic membrane transplantation.

**Case presentation:**

A 71-year-old Korean man developed bilateral herpetic keratitis one week after rapid tapering of systemic corticosteroid. He had been on high-dose oral corticosteroid and azathioprine therapy for six months for treatment of pemphigus foliaceus. Topical acyclovir ointment was prescribed. A week later, our patient's right eye had healed, but his left eye showed increased stromal edema with enlarged epithelial defects. He was prescribed oral acyclovir with topical broad-spectrum antibiotics applied to his left eye. The stromal edema cleared within a week but the epithelial defect remained unchanged. An amniotic membrane transplantation was performed on our patient's left eye, and his epithelial defect had totally healed three weeks later.

**Conclusions:**

Patients with autoimmune disease or who are on immunosuppressive therapy have a higher chance of developing bilateral herpetic keratitis. Although rare, the condition may be followed by unilateral neurotrophic keratitis. Rapid corticosteroid tapering may act as a triggering factor for viral infection or reactivation of herpes.

## Introduction

Herpes simplex keratitis is, in general, a unilateral disease, but bilateral occurrence has been reported in 1.3% to 10.9% of patients [1]. Bilateral herpetic keratitis is known to develop in patients with a compromised immune system, such as those with congenital immune deficiencies, atopy, long-term immunosuppression, or recipients of organ transplants [1-3].

Pemphigus is a group of autoimmune skin diseases with recurrent formation of acantholysis and chronic bullae within the epidermis. Pemphigus foliaceus (PF) is a subtype with a relatively mild clinical course. Early signs include eczematous erythema of the skin and mucous membrane erosions. Ocular involvement is often reported, with the majority of these lesions confined to the conjunctiva (for example, pseudomembranous conjunctivitis), but the cornea is seldom involved [4]. Herpes simplex virus infection often occurs during the course of pemphigus, but herpetic keratitis has been reported in only a few cases of pemphigus vulgaris and bullous pemphigoid [5,6]. We report a case of a patient with PF who developed bilateral herpetic keratitis immediately after rapid corticosteroid tapering. The disease followed an atypical course of neurotrophic keratitis.

## Case presentation

A 71-year-old Korean man was referred for both eye pain and epiphora starting a week previously. He had no history of other ocular disease. He had been diagnosed with PF six months before presentation, and had been started on immunosuppressive therapy with oral prednisolone (20 mg three times daily initially) and oral azathioprine (50 mg three times daily). His skin lesions had improved three weeks earlier, and since then his oral prednisolone dose had been tapered to 40 mg/day.

**Figure 1:**
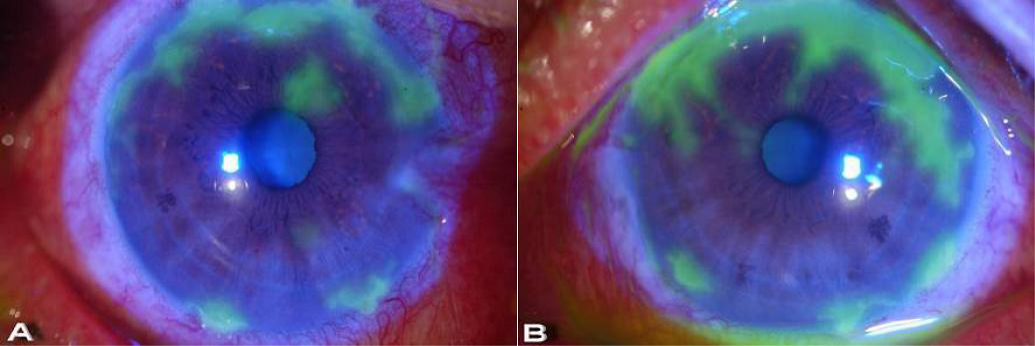
**Bilateral herpetic keratitis after steroid tapering**. Geographic epithelial defects in **(a) **the right eye and **(b) **the left eye.

On physical examination, our patient's best corrected visual acuities were 20/25 in the right eye and 20/50 in the left. Slit lamp examination showed conjunctival injection and geographical corneal ulcers (Figure 1). There was no inflammatory chamber reaction in either eye. Corneal esthesiometer (Cochet-Bonnet; Luneau Ophthalmology, Paris, France) measurements had decreased to 30 mm in both eyes.

We diagnosed our patient as having bilateral herpetic epithelitis, and prescribed topical 3% acyclovir (Herpecid ointment; Samil Pharmaceutical Co., Ltd., Seoul, Republic of Korea) ointment five times daily. Systemic antiviral agents were not used initially. After consulting with our dermatology department, we began tapering the oral prednisolone dose to 30 mg/day and replaced azathioprine with oral Cyclosporin A 100 mg twice a day.

**Figure 2:**
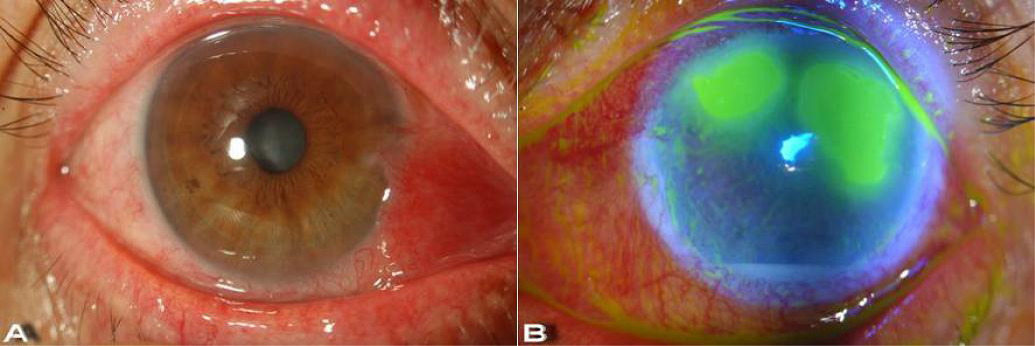
**Our patient one week after topical antiviral treatment**. **(a) **The right eye with healed epithelial defect. **(b) **Fluorescein-stained cornea of the left eye. The corneal epithelial defect has increased even with topical antiviral treatment, and hypopyon was detected.

A week after starting topical antiviral treatment, our patient's right eye had improved but the left eye had worsened. His best corrected visual acuities were 20/25 in the right eye but only hand motion in the left. On slit lamp biomicroscopy, the cornea and anterior chamber were clear in the right eye, but the left eye showed increased stromal edema and epithelial erosions (Figure 2). There was an aggravated chamber reaction (2+) and hypopyon 1 mm in size was detected in the left eye.

We started our patient on oral famciclovir 400 mg five times daily, with fortified topical antibiotics (10% cefazolin and 2% Gentamicin eye drops every two hours) added for the left eye, and the oral prednisolone dose tapered to 25 mg/day. Results from a corneal epithelial culture were negative for bacterial or fungal infection.

After three weeks of systemic and topical antiviral treatment, our patient's corneal edema, hypopyon and chamber reaction had resolved, but the corneal epithelial defect in the left eye remained unchanged. He was started on topical autoserum to be applied every two hours to the left eye.

**Figure 3:**
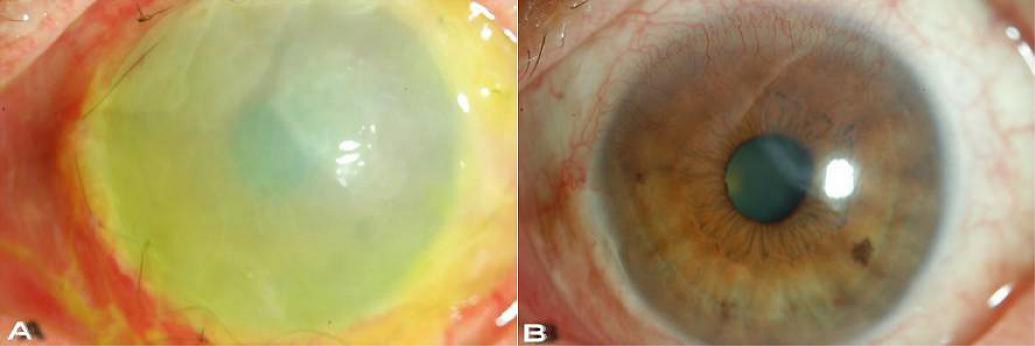
**Amniotic Membrane transplantation**. **(a) **Amniotic membrane transplantation on the left eye. **(b) **After three weeks, the amniotic membrane was removed, and the epithelial defect had totally healed.

We decided to perform an amniotic membrane transplantation over the corneal epithelial defect in the left eye. Before surgery, informed consent was obtained from our patient. Fresh frozen amniotic membrane (Cryopreserved; Bioland, Cheonan, Republic of Korea) was transplanted over the whole cornea with an on-lay technique, and was sutured to the conjunctiva with 10-0 nylon (Figure 3a). Post-operative treatment consisted of topical eye drops (0.5% levofloxacin (Cravit^®^); Santen Phamaceutical Co. Ltd, Osaka, Japan) four times daily and 3% acyclovir ointment (Herpecid) applied to our patient's left eye. His dose of oral prednisolone was tapered to 15 mg/day.

At three weeks after the amniotic membrane transplantation, the corneal epithelial defect in our patient's left eye had almost healed, and his dose of oral famciclovir was slowly tapered over the next three weeks, while the topical treatment with antibiotics and acyclovir ointment was continued in both eyes. After eight weeks of treatment, his best corrected visual acuities were 20/25 in both eyes, and the corneal epithelial defect had totally healed, leaving only a small area of mild subepithelial opacity (Figure 3b). Treatment with topical acyclovir ointment once daily was continued, with 0.5% carboxymethylcellulose eye drops (Refresh Plus^®^; Allergan, Inc., Irvine, CA, USA) every two hours. Oral prednisolone was slowly tapered (by 2.5 mg every two weeks) to a maintenance dose of 10 mg every other day, and oral Cyclosporin A was maintained at 150 mg twice daily. The systemic corticosteroid was slowly tapered (by 2.5 mg every two weeks) and our patient was kept on a maintenance dose. At follow-up examination a year later, there was no sign of recurrent infection, and his best corrected visual acuities were maintained in both eyes. There was also no sign of exacerbation of any skin lesions.

## Discussion

In the management of exacerbation periods of PF, the treatment of choice is high-dose corticosteroid combined with immunomodulative drugs such as azathioprine or Cyclosporin A and supplementary antibiotics [4]. Patients with pemphigus often present with herpes simplex virus infection, which is generally regarded as an opportunistic infection, because these patients are usually on long-term immunosuppressive therapy. Ocular involvement often occurs, but most of the reported cases have been confined to conjunctival lesions [4].

Our patient with PF had been on long-term immunosuppressive treatment for over six months. As his skin lesions had improved, the oral prednisolone dose was tapered to 20 mg/day over a week. Bilateral herpetic keratitis developed immediately after he had reached 20 mg/day. Considering the temporal correlation of the corticosteroid tapering and the symptom development, and the fact that there were no blistering lesions on the cornea, we suggest that the herpetic keratitis was not a complication of the PF itself but was a type of opportunistic infection. This may have been a latent infection reactivated by rapid tapering of the systemic corticosteroid, as both steroid use and rapid tapering are known to be risk factors for recurrent herpetic keratitis [7-9]. As in our patient, rapid systemic steroid tapering might exacerbate inflammatory reactions after primary opportunistic infections or might trigger reactivation of previous latent infections.

Long-term oral antiviral prophylaxis has demonstrated a significant decrease in recurrence of all forms of herpetic eye disease [10]. In our patient, systemic antiviral agents were used for no longer than nine weeks, but there was no sign of recurrence over the following year.

Our case is interesting because the disease progression produced unusual features in both eyes. Bilateral herpetic keratitis usually follows a symmetric course in both eyes, but this was not true for our patient [2]. After initial topical antiviral ointment therapy only the right eye improved, whereas the left had worsened. Where the right eye was limited to a simple epithelitis, the left eye progressed to an additional endothelitis or sterile ulcer. This kind of asymmetry is not common in herpes keratitis. Oral acyclovir was prescribed to control stromal inflammation, and the condition was successfully managed [8]. Such atypical findings might be related to the underlying systemic condition of our patient, as patients who are immunocompromised are apt to show atypical features during disease course [3].

In herpetic keratitis, the use of amniotic membrane transplantation has been reported in a case of acute necrotizing herpetic keratitis [11,12]. As in our patient, amniotic membrane transplantation is useful in encouraging re-epithelization of persistent epithelial defects and reducing stromal inflammation after remission of the acute stage of herpetic keratitis, and should be considered as a mainstay of treatment of neurotrophic herpetic keratitis.

## Conclusions

Patients with pemphigus on long-term corticosteroid treatment are immunosuppressed, and therefore have a higher chance of opportunistic infection with herpetic keratitis. Herpetic keratitis involving both eyes is more often seen in patients who are immunocompromised, and unusual features may develop [3]. Rapid steroid tapering may be a risk factor for sudden disease activation. These patients should receive regular and thorough ocular examinations especially if they are on immunosuppressive treatment, and clinicians should be aware of such atypical features.

## Consent

Written informed consent was obtained from the patient for publication of this case report and any accompanying images. A copy of the written consent is available for review by the Editor-in-Chief of this journal.

## Competing interests

The authors declare that they have no competing interests.

## Authors' contributions

HKY and YKH interpreted our patient's medical history and were major contributors to writing the manuscript. WRW and JWK performed the clinical observation and treatment of our patient. JHL revised the manuscript critically for important intellectual content. All authors read and approved the final manuscript.
